# Artifacts in Cone-Beam Computed Tomography of a Post and Core Restoration: A Case Report

**Published:** 2012-06-01

**Authors:** Masoud Parirokh, Karim Ardjomand, Hamed Manochehrifar

**Affiliations:** 1. Oral and Dental Diseases Research Center, Kerman University of Medical Sciences, Kerman, Iran; 2. Private practice, Kerman, Iran; 3. Department of Endodontics, Dental School, Kerman University of Medical Sciences, Kerman, Iran

**Keywords:** Artifacts, Cone-Beam Computed Tomography, Endodontic, Root Canal Therapy, Radiography

## Abstract

Cone-beam computed tomography (CBCT) has been accepted as a useful tool for diagnosis and treatment in endodontics. Despite a growing trend toward using CBCT in endodontic practice the CBCT images should be interpreted carefully. This case report presents a case that showed radiolucency inside and around a tooth which was free of pathologic changes under a dental operative microscope and conventional radiographs. A male patient was referred to an endodontic office for evaluation of radiolucency inside and around tooth #21 in his CBCT images. The post and crown over the tooth was removed and the tooth was observed under a dental operative microscope. Clinical examination as well as direct observation under a dental operative microscope showed no pathological lesions inside and around the tooth. The misdiagnosis was based on an artifact on CBCT. Despite the advantages of CBCT images as a great radiographic aid in endodontic practice, in the presence of metallic structures such as post and core the images should be interpreted with caution.

## Introduction

The introduction of cone-beam computed tomography (CBCT) has made a revolutionary impact on the diagnosis of complicated cases in endodontic practice. Several investigations and case reports have shown that CBCT is a useful device for diagnosis of periapical lesions, detection of vertical fracture, detection of invasive cervical resorption, investigating root canal anatomy and endodontic mishaps [1][2][3][4][5][6][7].

However, CBCT technology has not been recommended as a routine radiographic aid for all patients in need of endodontic treatment [8]. In a recently published position paper by the American Association of Endodontists (AAE) and the American Academy of Oral and Maxillofacial Radiology (AAOMR) [8] the following conditions have been recommended for the use of CBCT in endodontics: identification of the presence of accessory canals in suspected teeth, identification of root canal anomalies and determining root curvature, diagnosis of periapical lesion in complicated case that are associated with atypical sign and symptoms and without presence of periapical pathosis in their conventional radiographic image or presence of nonodontogenic reasons, in the diagnosis of the extent of nonodontogenic origin pathosis, assessment of intra- or postoperative root canal treatment complications (broken endodontic instruments, perforations, overextended root canal fillings, and presence of calcified root canals). They have also been recommended for the diagnosis and management of dental trauma, diagnosis and management of internal and external resorption whenever possible, assessment of the proximity of anatomical structures to the root apices before performing endodontic surgical treatment, and of edentulous ridge for the placement of dental implant [8]. Despite the numerous advantages of using CBCT as a diagnostic aid in clinical practice, some limitations have been described [9][10] particularly when an intracanal metallic post is used [9]. Sometimes metallic artifacts in conjunction with intracanal post may be misdiagnosed as a perforation or destructive lesion around a suspected root. Photons with a wide range of energy produce x-ray beam. As low energy photons are absorbed quickly during passing x-ray through an object higher energy photons get stronger and therefore two types of artifact may form: i) cupping artifacts and ii) streak and dark bands artifacts. The latter artifact can form when a metallic structure has been in or on the body of the patient in the scan field [11].

This report presents a case with radiolucency inside and around a mandibular premolar tooth that was clinically and radiographically free of pathologic lesion.

## Case Report

**Figure 1:**
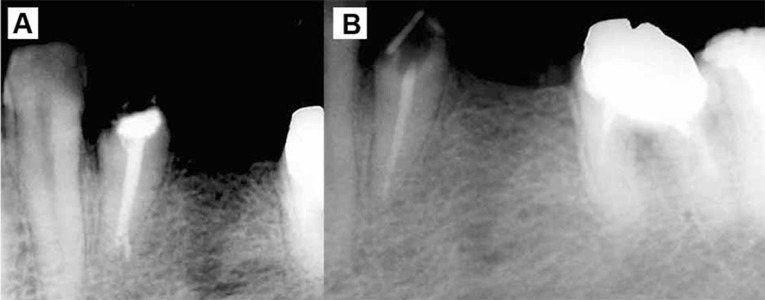
A) Radiographic image of the tooth #21 before retreatment; B) Radiographic image of tooth #21 after retreatment 11 years before taking CBCT image

A 56-year-old male was referred for evaluation of his left mandibular first premolar tooth (#21). His past dental history showed that the tooth had been retreated 11 years previous (Figure 1) followed by a post and core restoration. A fixed prosthetic restoration that employed teeth #18 and #21 as abutments was made at the same time.

**Figure 2 A-C:**
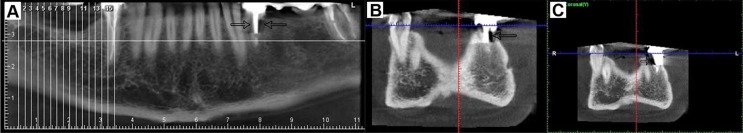
Cone-beam computed tomography images from the tooth #21 shows radiolucent lesion inside and around the tooth (open arrows)

**Figure 3:**
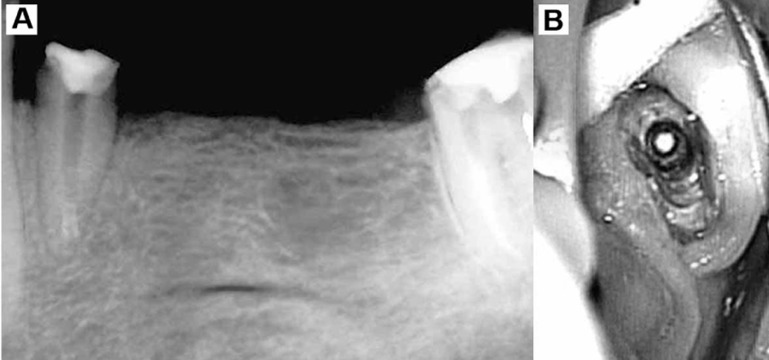
A) Periapical radiographic image which was taken following bridge and post removal; B) Photographic image of the tooth #21 taken with a dental operative microscope camera (×1.6)

The patient’s chief complaint when referred to a periodontist office was fracture of the ceramic of the bridge which he wished to replace with two single tooth implants in his edentulous ridge. A CBCT was ordered by the periodontist and during the evaluation of the images a radiolucent lesion was detected around the tooth #21 at both coronal and sagittal views (Figure 2). The bridge and subsequently the post were removed. Then a periapical radiograph was taken (Figure 3). The patient was then referred to our office for further evaluation and consultation. The tooth was examined under a dental operative microscope (DOM; Carl Zeiss, Oberkochen, Germany) for any sign of resorption or perforation. No sign of perforation was observed either during root observation by DOM or the periapical radiographic image (Figure 3 ). Pperiodontal status of the tooth was evaluated and was found to be within normal limits. The tooth was diagnosed as being sound with only a CBCT artifact. The patient was advised to have a root canal retreatment of the tooth before placing a new post and crown because of the inadequate amount of remaining gutta-percha in the root canal following previously performed post space preparation. A recent article introduced map reading for overcoming this shortcoming [9]; however, in the present case map reading could not help the examiners to distinguish between the presence of sound intact tooth and periodontal structure. In fact, map reading from the coronal view illustrated severe damage of the root structure of the tooth.

## Discussion

This case report shows how CBCT can cause artifacts in teeth with intracanal posts; this is a significant disadvantage of CBCT imaging technique. Presence of a metallic artifact in the field of scan will produce streaks and dark bands because a portion of the beam passes through an object at one position making it harder in the other tube positions. Several techniques and equipments have been described to overcome this predicament: filtration [12], calibration [11], linearization [13] and correction beam hardening software [11]. A recent investigation demonstrated that the presence of metallic post reduced both sensitivity and specificity of CBCT for the detection of horizontal root fracture [14].

In our study, the radiolucent lesion around the tooth root was at the coronal third and therefore, should have had some negating effect on the periodontal status of the tooth during clinical/radiographic examination. However, as the pocket depth was normal and so was the periodontal structure and texture, we can conclude that the tooth and its surrounding structures were free of any pathologic changes.

In the present case, DOM was used as a diagnostic aid for evaluating the root structure. Direct view by DOM showed no discrepancy at the post space.

There is no doubt that CBCT is a valuable aid in endodontic practice; however, AAE and AAOMR have stated that it should not be ordered routinely for all patients in need of endodontic treatment [8]. In fact, the potential risks and benefits of CBCT for each patient should be fully assessed based on both his/her conventional radiograph(s) and clinical signs and symptoms. In the present case, a CBCT was ordered for the placement of implants in the patient’s edentulous ridge which is recommended by the AAE and AAOMR [8].

If the CBCT is retaken after removal of the post and core, the radiolucency around and inside the tooth will disappear showing that it was an artifact. However, assessment of the possible risks and benefits of retaking the CBCT image as well as ethical concerns contraindicated this step. Moreover, Shemesh et al. have reported no significant difference between CBCT and digital periapical radiography for detection of root perforation [15].

Both coronal and sagittal views of the tooth CBCT falsely emulated root perforation image contraindicating the periapical radiograph and clinical findings of the tooth. In our case study, an inappropriate post-space preparation had left an empty space between the post and the root canal filling material which could have mislead the clinician in believing that the CBCT showed a real lesion.

## Conclusions

In conclusion, the present case has shown that CBCT images should be interpreted with caution especially with post and core restorations.
